# Feasibility of weekly patient-reported symptom monitoring using patients' own smartphones in outpatient cancer chemotherapy: the SMART-PRO study

**DOI:** 10.3389/fdgth.2026.1792647

**Published:** 2026-06-08

**Authors:** Yutaka Sugawara, Momoko Kobayashi, Eri Mannoji, Tomoya Saika, Yuki Kado, Michiko Yamazaki, Tohru Takebe, Hajime Higuchi

**Affiliations:** 1 Department of Clinical Oncology, International University of Health and Welfare, Narita Hospital, Chiba, Japan; 2 Patient Engagement Frontier Medical Science, KAKEHASHI Inc., Tokyo, Japan; 3 Graduate School of Medical and Pharmaceutical Sciences, Chiba University, Chiba, Japan; 4 Department of Hematology, Nippon Medical School, Tokyo, Japan

**Keywords:** bring-your-own-device, electronic patient-reported outcomes, older adults, outpatient oncology, PRO-CTCAE, symptom monitoring

## Abstract

Electronic patient-reported outcome (ePRO) systems using the Patient-Reported Outcomes version of the Common Terminology Criteria for Adverse Events (PRO-CTCAE) can improve symptom monitoring, but the feasibility of implementing such systems with a bring-your-own-device (BYOD) approach in routine oncology practice, particularly among older adults, is not well established. We conducted a single-arm prospective observational study in Japan to evaluate weekly BYOD-based ePRO monitoring of adverse events in outpatients receiving chemotherapy. Twenty-eight patients used smartphones to complete weekly PRO-CTCAE/CTCAE-based questionnaires for 12 weeks. Alerts were automatically generated when severity or frequency thresholds were met. Feasibility outcomes were weekly ePRO response rates and the distribution of alert-generating symptoms; HRQoL (EORTC QLQ-C30) was assessed at baseline, week 6, and week 12. The mean age was 66.7 years and most patients had stage IV disease. The weekly ePRO response rate ranged from 75% to 93% in the overall cohort and remained at or above 75% in patients aged ≥70 years. Pain-related items most frequently triggered alerts, followed by numbness and tingling, rash, fatigue, and skin-related disorders. The EORTC QLQ-C30 global health status score increased numerically from 54.8 at baseline to 63.5 at week 6 and 63.4 at week 12, with no marked deterioration observed over 12 weeks. These findings indicate that weekly PRO-CTCAE/CTCAE-based ePRO monitoring using a BYOD approach is feasible in routine outpatient oncology care, including among older adults who use smartphones. This study provides preliminary evidence for BYOD-based ePRO implementation in this underexplored population.

## 1 Introduction

Adverse events (AEs) are common during cancer drug therapy, and incorporating patients' perspectives through patient-reported outcomes (PROs) have become increasingly important. The Patient-Reported Outcomes version of the Common Terminology Criteria for Adverse Events (PRO-CTCAE), developed by the U.S. National Cancer Institute, provides a standardized way to assess treatment-related AEs from the patient's perspective and has demonstrated reliability and validity in oncology settings (1). In oncology, PRO-based symptom monitoring has been highlighted as a means of improving clinical care and patient–clinician communication (2), and randomized trials such as the PRO-TECT study have shown that electronic PRO (ePRO)–based symptom monitoring can maintain or improve health-related quality of life (HRQoL), reduce emergency visits and hospitalizations, and even improve survival compared with usual care (3, 4). More recently, randomized controlled trials such as PRO-DUCE and the NRG-BR004 PRO-CTCAE substudy have evaluated the feasibility of ePRO-based symptom monitoring using systems that incorporate PRO-CTCAE items in clinical trial settings (5, 6).

However, most of this evidence comes from clinical trial settings that rely on study-provided devices or dedicated web-based systems with intensive support from study staff. In parallel, bring-your-own-device (BYOD) approaches for ePRO data collection, in which patients use their own smartphones or tablets, have shown acceptable feasibility, patient acceptance, and measurement equivalence compared with study-provided devices in younger or mixed-age adult populations (7, 8). In Japan, however, a large proportion of patients with cancer are older adults, and older patients generally have lower rates of smartphone and internet use and lower eHealth literacy than younger patients (9, 10), raising concerns about the usability and burden of BYOD-based ePRO monitoring in this context. Consequently, the feasibility of BYOD-based ePRO monitoring for older adults with cancer in routine clinical practice has not been sufficiently established.

Therefore, we conducted a single-arm prospective observational study in the outpatient chemotherapy setting at a Japanese hospital to implement and evaluate weekly ePRO monitoring of treatment-related AEs using PRO-CTCAE/CTCAE with a BYOD approach.

## 2 Materials and methods

### 2.1 Study design and setting, and registration

The Systematic Monitoring of Adverse Reactions and Treatment using PRO (SMART-PRO) study was a single-arm prospective observational study conducted at the International University of Health and Welfare Narita Hospital, Japan. The enrollment period was from May 7 to October 11, 2024, and participants were followed for 12 weeks after enrollment. The target enrollment was 30 patients, and no sample size calculation was performed for this proof-of-concept (PoC) study. The study was registered in the UMIN Clinical Trials Registry (UMIN000055138).

### 2.2 Participants

Eligible participants were outpatients (aged ≥20 years) receiving anticancer chemotherapy at this hospital. The key inclusion criteria were a diagnosis of malignancy; ECOG performance status 0–2; physician-estimated prognosis of ≥6 months; and ability to continuously operate a smartphone (bring your own device; BYOD), as judged by the treating physician based on their routine use of their own devices. Exclusion criteria were patients clearly unable to use touch-screen electronic devices for data entry; patients clearly unable to self-report symptoms due to mental illness or cognitive impairment; patients participating in other PRO studies during the same period that would interfere with ePRO entry in this study; and patients deemed inappropriate by the treating physician for clinical or psychosocial reasons.

### 2.3 ePRO-based adverse event monitoring procedure

Adverse event (AE) monitoring was conducted using Pocket Musubi (KAKEHASHI Inc., Tokyo, Japan), a patient follow-up application that operates on the LINE platform (LY Corporation, Tokyo, Japan), a widely used mobile messaging application in Japan (11). Participants installed LINE and Pocket Musubi on their own smartphones and completed PRO questionnaires once weekly for 12 weeks (Supplementary Figure S1).

Weekly questionnaires were based on the PRO-CTCAE (version 1.0, Japanese version) (12, 13) and the Japanese translation of CTCAE v5.0 (CTCAE v5.0–JCOG, MedDRA v20.1/MedDRA/J v25.1) (14). Although CTCAE is conventionally clinician-rated, in this study we incorporated selected CTCAE items into the patient questionnaire to capture patient-reported severity for monitoring purposes. To support feasibility in routine practice and reduce patient burden, the questionnaire content was restricted to 35 items derived from PRO-CTCAE/CTCAE through discussion with the clinical team, prioritizing common, clinically actionable symptoms (Table 1). For severity and frequency items, responses were coded from 0 to 4 according to the original response categories; the death category was not included given the patient-reported nature of the assessments. For presence/absence items, symptoms were recorded as present or absent.

**Table 1:** Symptoms included in the ePRO questionnaire based on PRO-CTCAE and CTCAE terms.

Symptoms from PRO-CTCAE	Attribute assessed	Symptoms from CTCAE	Attribute assessed
Mouth/throat sores	Severity	Mucositis oral	Severity
Taste changes	Severity	Dysgeusia	Severity
Decreased appetite	Severity	Anorexia	Severity
Nausea	Frequency	Nausea	Severity
Vomiting	Frequency	Vomiting	Severity
Vomiting	Severity	Constipation	Severity
Constipation	Severity	Diarrhea	Severity
Diarrhea	Frequency	Dyspnea	Severity
Shortness of breath	Severity	Edema limbs	Severity
Swelling	Frequency	Rash acneiform/Rash maculo-papular	Severity
Rash	Presence/Absence	Pruritus	Severity
Acne	Severity	Palmar-plantar erythrodysesthesia syndrome	Severity
Skin dryness	Severity	Skin	Presence/Absence
Itching	Severity	Nail loss	Severity
Hand–foot syndrome	Severity	Pain	Severity
Nail loss	Presence/Absence	Fatigue	Severity
Numbness and tingling	Severity		
General pain	Frequency		
Fatigue	Severity		

Symptom names are based on the PRO-CTCAE item library (Japanese version) and CTCAE v5.0–JCOG terminology; full item wording is not reproduced.

Questionnaires were delivered to participants every Wednesday via the application. Responses were aggregated into a weekly report, in which alert-triggering AEs were highlighted. Alert thresholds were defined as responses of 3 or 4 for severity and frequency items and “present” for presence/absence items, informed by previous ePRO monitoring studies (3, 4). The report was stored every Friday in a designated secure hospital folder with restricted access. Treating physicians were expected to review the report as needed during outpatient visits (Supplementary Figure S2).

### 2.4 Outcomes

The study evaluated both feasibility outcomes and PROs over the 12-week follow-up period. Feasibility was assessed using the weekly ePRO response rate and the weekly alert issuance rate. No feasibility threshold was prespecified *a priori* because this exploratory PoC study aimed to describe the use of BYOD-based ePRO monitoring in routine practice. Health-related quality of life (HRQoL) was assessed using the validated Japanese version of the EORTC QLQ-C30 (version 3.0) (15, 16). Questionnaires were administered in paper form at baseline, weeks 6, and week 12. Patient experience was assessed in paper form at weeks 6 and 12 using the Japanese version of the CAHPS Clinician & Group Adult Survey 3.0 (CG-CAHPS) (17). The Japanese CG-CAHPS is an 18-item measure of patient experience in ambulatory care, comprising four composite domains (Access, Provider Communication, Care Coordination, and Office Staff) and a Provider Rating. Scores are converted to a 0–100 scale, with higher scores indicating better patient experience, and were analyzed descriptively as an exploratory outcome.

Weekly ePRO response rate was defined as the proportion of completed questionnaires among all questionnaires delivered each week (denominator: questionnaires delivered in that week; numerator: completed questionnaires in that week). Response rates were summarized for the overall cohort and for a prespecified older subgroup (aged ≥70 years). Weekly alert issuance rate was calculated among completed questionnaires only and defined as the proportion of responses that contained at least one alert-triggering item (denominator: completed questionnaires in that week; numerator: completed questionnaires with ≥1 alert-triggering item in that week).

### 2.5 Statistical analysis

Baseline participant characteristics were summarized using mean ± standard deviation for continuous variables and frequency (percentage) for categorical variables. Weekly ePRO response rates and weekly alert issuance rates were summarized as proportions over time, and 3-week moving averages of weekly ePRO response rates, defined as the mean of the preceding, current, and following weekly values, were calculated for the overall cohort and for the subgroup aged ≥70 years. HRQoL (EORTC QLQ-C30) and patient experience (CG-CAHPS) scores were summarized descriptively at each scheduled time point. No formal hypothesis testing was planned for this feasibility-focused report. No imputation was performed for missing data. All statistical analyses were performed using R version 4.3.2 (R Foundation for Statistical Computing, Vienna, Austria).

### 2.6 Ethics

The study was approved by the Ethics Committee of the International University of Health and Welfare (approval number: 21-Im-066). The study was conducted in accordance with applicable ethical standards and regulations.

## 3 Results

### 3.1 Patient characteristics

A total of 28 patients were enrolled in the SMART-PRO study. The mean age was 66.7 years (standard deviation 10.6), and 18 patients (64.3%) were male. Most patients had stage IV disease (92.9%), and colorectal and gastric cancers were the most frequent primary sites. Baseline characteristics are summarized in Table 2.

**Table 2:** Patient characteristics.

Characteristic	*N* = 28
Age, years, mean ± SD	66.7 ± 10.6
≥70 years	13 (46.4)
Male	18 (64.3)
Tumor site	
Colorectal cancer	6 (21.4)
Gastric cancer	15 (53.6)
Lung cancer	1 (3.6)
GIST	1 (3.6)
Others	5 (17.9)
Stage	
III	2 (7.1)
IV	26 (92.9)
Chemotherapy regimen	
CapeOX-based	9 (32.1)
FOLFIRI-based	4 (14.3)
FOLFOX-based	4 (14.3)
Others	11 (39.3)
Molecular-targeted agents	
Bevacizumab	8 (28.6)
Ramucirumab	5 (17.9)
Panitumumab	4 (14.3)
Sunitinib	1 (3.6)
Trastuzumab	1 (3.6)
Encorafenib/Cetuximab	1 (3.6)
Others	8 (28.6)

Data are presented as *n* (%) unless otherwise indicated. GIST, gastrointestinal stromal tumor; CapeOX, capecitabine plus oxaliplatin; FOLFIRI, infusional 5-fluorouracil, leucovorin, and irinotecan; FOLFOX, infusional 5-fluorouracil, leucovorin, and oxaliplatin.

### 3.2 Feasibility of BYOD-based ePRO monitoring

**Figure 1:**
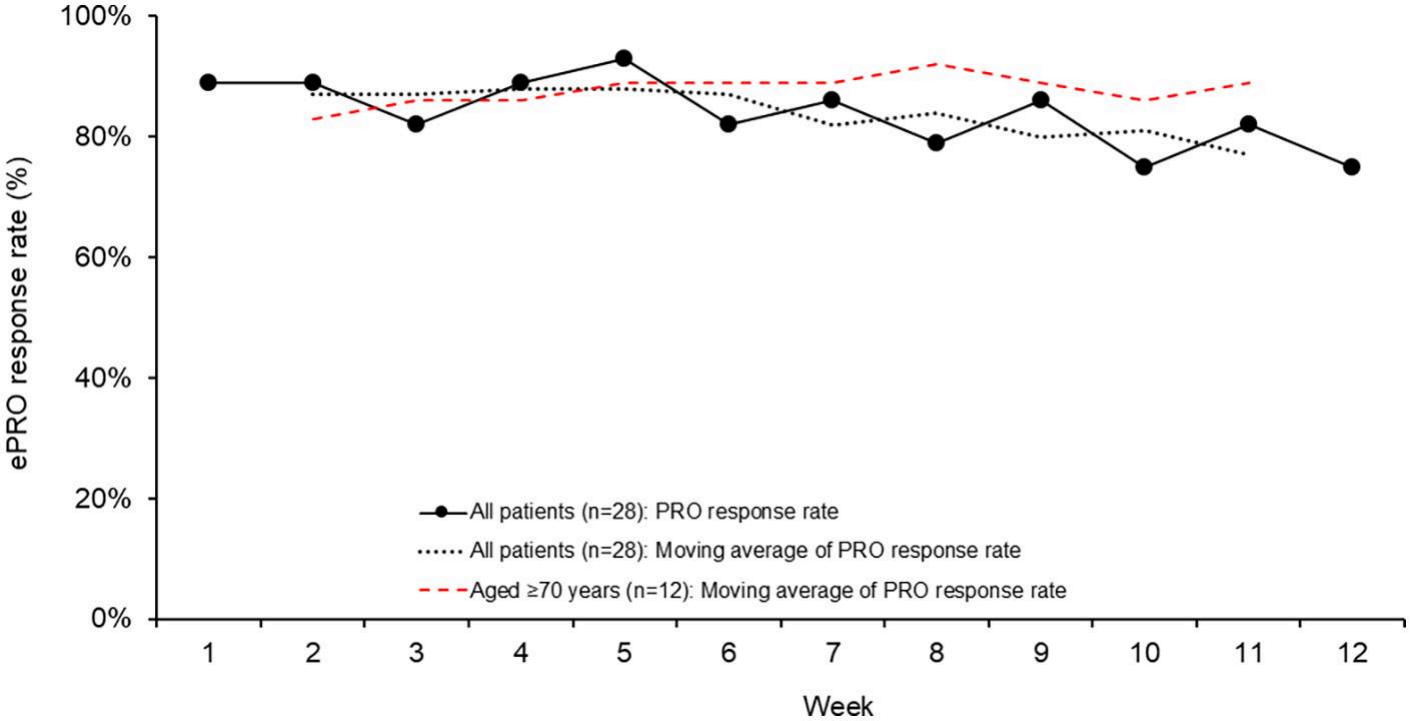
Weekly ePRO response rates over the 12-week observation period. The solid line shows the weekly response rate for all patients (*n* = 28). The dotted line indicates the 3-week moving average of the response rate for all patients, and the dashed red line indicates the moving average for patients aged ≥70 years (*n* = 12).

Over the 12-week observation period, the weekly ePRO response rate in the overall cohort was 89% at week 1 and ranged from 75% to 93%. The 3-week moving average ranged from 77% to 88% overall and from 83% to 92% in patients aged ≥70 years (Figure 1 and Supplementary Table S1).

**Figure 2:**
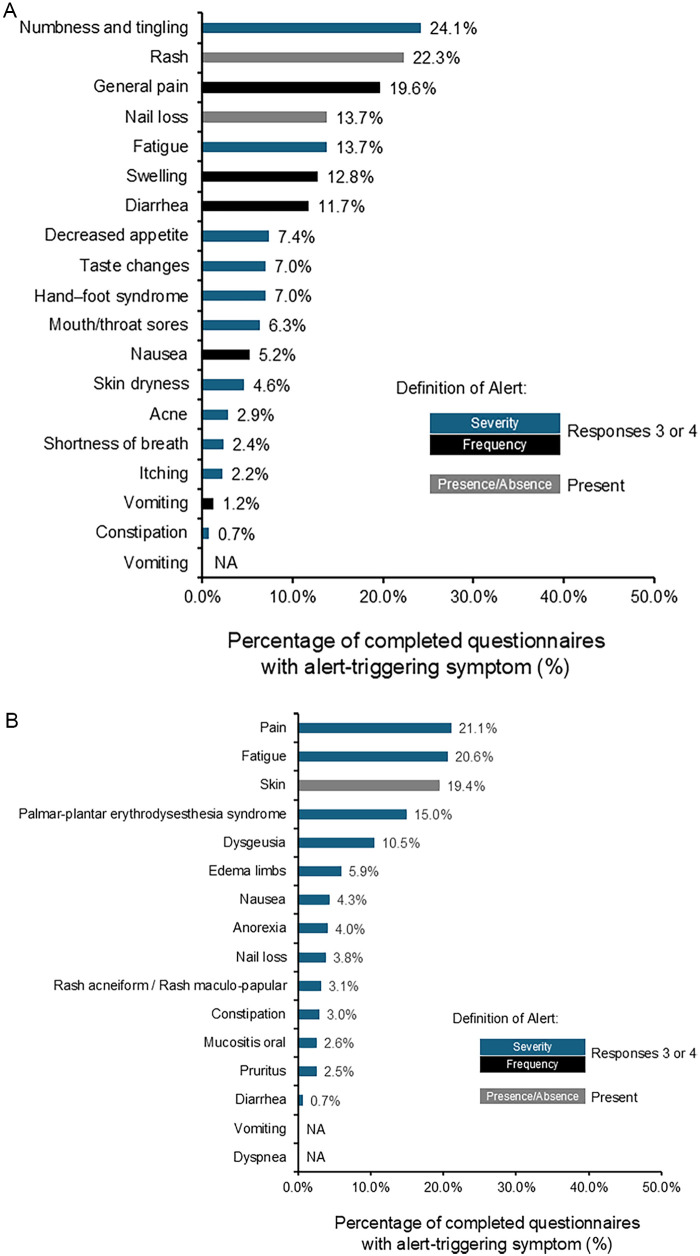
Distribution of alert-triggering symptoms captured by the ePRO system. **(A)** Mean weekly alert rates for symptoms based on PRO-CTCAE. **(B)** Mean weekly alert rates for symptoms based on CTCAE. In both panels, bars represent the mean weekly proportion of completed questionnaires over the 12-week observation period in which each symptom met the prespecified alert threshold (responses of 3 or 4 for severity or frequency, or present for presence/absence items). NA indicates that no alert was observed for that symptom during the study period.

Regarding alerts, numbness and tingling, rash, and general pain were the most frequent alert-triggering symptoms based on PRO-CTCAE, meeting the alert threshold in 24.1%, 22.3%, and 19.6% of all completed questionnaires, respectively. When mapped to CTCAE terms, the most frequent alert categories were pain (21.1%), fatigue (20.6%), and skin-related disorders (19.4%) (Figures 2A,B). The patient-level distribution of the total number of alerts over 12 weeks is shown in Supplementary Figure S3.

Clinician use of the weekly ePRO reports was not quantified, and no routine use was identified during the observation period.

### 3.3 Exploratory PROs

The EORTC QLQ-C30 global health status score (0–100 scale) was 54.8 at baseline (*n* = 26), 63.5 at week 6 (*n* = 16), and 63.4 at week 12 (*n* = 23) among patients with available data, showing a numerical increase over the 12-week period. Among patients with both baseline and week 12 assessments (*n* = 22), 9 (40.9%) had an improvement of ≥5 points and 8 (36.4%) had a worsening of ≥5 points in global health status.

The evaluated CG-CAHPS scores were generally high, remaining above 80 on the 0–100 scale at both week 6 and week 12, but did not show a consistent change during the observational period. CG-CAHPS results were interpreted descriptively as exploratory outcome.

## 4 Discussion

This study evaluated the feasibility of weekly ePRO monitoring based on PRO-CTCAE and CTCAE, using a BYOD approach in routine outpatient chemotherapy. Over the 12-week observation period, weekly ePRO response rates were maintained (week 1: 89%; range: 75%–93%) in the overall cohort, with similar rates in patients aged ≥70 years. Alerts occurred frequently for clinically important symptoms such as pain, neuropathic symptoms, dermatologic symptoms, and fatigue, indicating that the BYOD-based ePRO system captured relevant symptom burden in real-world practice. The EORTC QLQ-C30 global health status score showed a numerical increase, and we did not observe marked deterioration over 12 weeks, suggesting that this weekly ePRO schedule did not impose an excessive burden on patients. Together, these findings indicate that weekly BYOD-based ePRO monitoring is acceptable and feasible in routine oncology care, including for older adults who regularly use smartphones.

Among older patients with cancer, smartphone and internet use and eHealth literacy are generally lower than in younger populations, and factors such as anxiety about device operation and declines in visual or cognitive function have been reported as potential barriers to completing ePRO assessments; therefore, cautious views have been expressed regarding the implementation of BYOD-based ePRO monitoring in older adults (10, 18–20). To address these concerns, we delivered ePRO questionnaires via Pocket Musubi on the LINE platform, which is widely used even among older age groups (e.g., >90% among people in their 60s) (11). Under these conditions, both the overall cohort and patients aged ≥70 years maintained weekly ePRO response rates of 75% or higher in all weeks. As an exploratory PoC study, no feasibility threshold was prespecified *a priori*; however, we used 75% as a pragmatic reference point, informed by a recent PRO-CTCAE trial that defined participant-level compliance as submission of ≥75% of scheduled weekly assessments over 12 weeks (6). Our findings suggest that weekly ePRO monitoring based on standardized PRO-CTCAE/CTCAE items can be feasibly implemented using a BYOD approach in routine clinical practice among older patients with cancer who regularly use smartphones.

Conversely, the use of ePRO reports by physicians was limited. This finding highlights the need for workflow integration that enables physicians to easily access ePRO information for clinical decision-making. Workflow integration remains an important barrier to the effective use of ePRO data in routine clinical practice and requires further attention in future studies.

This study has several limitations. First, this was a small (*n* = 28), single-center, single-arm observational study conducted in Japan without a comparator group, limiting generalizability and precluding causal inference regarding the effects of BYOD-based ePRO monitoring. In particular, response rates may differ across domestic and international settings. Second, only participants who were able to use smartphones on a continuous basis were enrolled, which may have introduced selection bias toward smartphone-literate participants and limits generalizability to those with lower eHealth literacy. Third, most participants had stage IV disease, limiting assessment of engagement in other disease stages. Related ePRO studies also suggest that adherence may vary by disease burden or stage (21). Fourth, we used a pragmatically selected set of 35 PRO-CTCAE/CTCAE items and corresponding alert thresholds, rather than items chosen through a formal, standardized process; different item sets or thresholds might yield different alert frequencies and distributions. Fifth, because CTCAE is conventionally clinician-rated, comparability with studies using clinician-assessed CTCAE may be limited. Sixth, the 12-week observation period was insufficient to assess longer-term sustainability, including potential engagement decay beyond 3 months. Longer prospective studies are warranted, and we are conducting studies with ≥6 months of follow-up (PHARE-ONC 01 Study, UMIN000056007; PHARE-ONC 02 RT Study, UMIN000056035; PHARE-HF Study, UMIN000055597).

Despite these limitations, our findings indicate that weekly BYOD-based ePRO monitoring using standardized PRO-CTCAE/CTCAE items can be implemented with high response rates in routine outpatient oncology practice, including among older adults who regularly use smartphones. However, limited clinician use of ePRO reports underscores that future work must pair BYOD-based approaches with better integration of ePRO data into routine clinical workflows, particularly electronic medical records, to ensure that patient-reported information is effectively used in care.

## 5 Conclusion

Weekly BYOD-based ePRO monitoring using PRO-CTCAE/CTCAE was feasible in routine outpatient oncology practice, with high response rates including older adults who regularly use smartphone. However, limited clinician use of ePRO reports highlights the need for better integration of patient-reported data into routine clinical workflows in future implementations.

## Data Availability

The datasets generated and analyzed in this study are not publicly available because they contain individual-level clinical information obtained through a proprietary ePRO platform and cannot be shared outside the study team. Requests to access the datasets should be directed to Tomoya Saika t.saika@kakehashi.life.
