# Supplementary material for: Feasibility of weekly patient-reported symptom monitoring using patients' own smartphones in outpatient cancer chemotherapy: the SMART-PRO study
Source: Front Digit Health. 2026 Jun 8;8:1792647. doi: 10.3389/fdgth.2026.1792647 (PMC13284108; doi:10.3389/fdgth.2026.1792647)
Supplement: Supplementary file 1 [file Datasheet1.docx]

Supplementary Material

**Supplementary Table S1.**

|  | All patients | | Patients aged ≥70 years | |
| --- | --- | --- | --- | --- |
| Week | ePRO response rate | Moving average of ePRO response rate | ePRO response rate | Moving average of ePRO response rate |
| 1 | 89.0% | ‐ | 83.0% | ‐ |
| 2 | 89.0% | 86.7% | 92.0% | 83.3% |
| 3 | 82.0% | 86.7% | 75.0% | 86.3% |
| 4 | 89.0% | 88.0% | 92.0% | 86.3% |
| 5 | 93.0% | 88.0% | 92.0% | 89.0% |
| 6 | 82.0% | 87.0% | 83.0% | 89.0% |
| 7 | 86.0% | 82.3% | 92.0% | 89.0% |
| 8 | 79.0% | 83.7% | 92.0% | 92.0% |
| 9 | 86.0% | 80.0% | 92.0% | 89.0% |
| 10 | 75.0% | 81.0% | 83.0% | 86.0% |
| 11 | 82.0% | 77.3% | 83.0% | 88.7% |
| 12 | 75.0% | ‐ | 100.0% | ‐ |

**Supplementary Figure S1.** Study timeline and schedule of ePRO, HRQoL, and patient experience assessments.


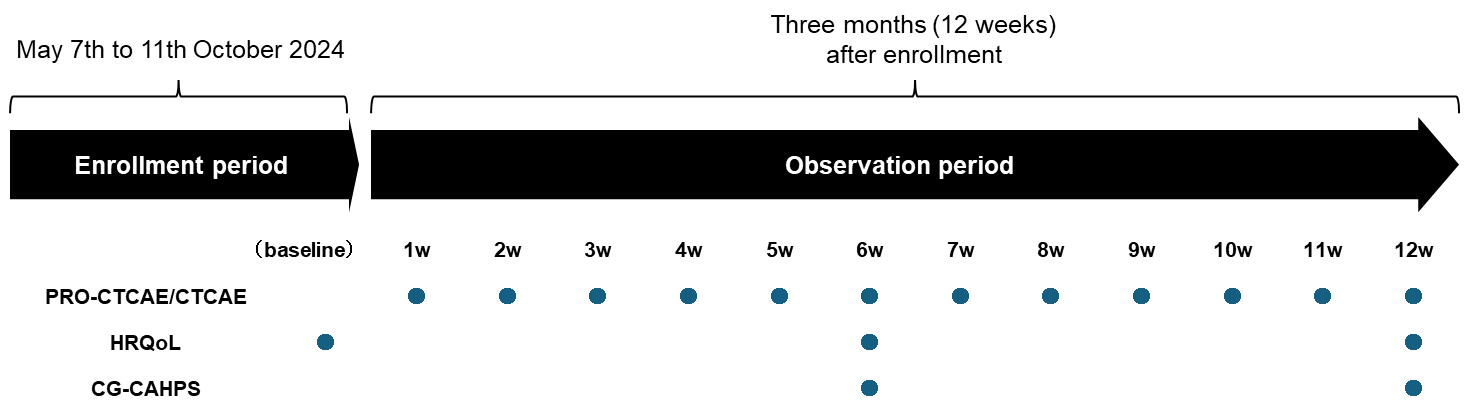


The enrollment period was from May 7 to October 11, 2024. Each participant was followed for 12 weeks after enrollment. PRO-CTCAE/CTCAE ePRO assessments were administered weekly during the 12-week observation period. Health-related quality of life (HRQoL) was assessed with the EORTC QLQ-C30 at baseline, week 6, and week 12, and patient experience was assessed with the CG-CAHPS at weeks 6 and 12.

**Supplementary Figure S2.** Workflow of the BYOD-based ePRO monitoring system.


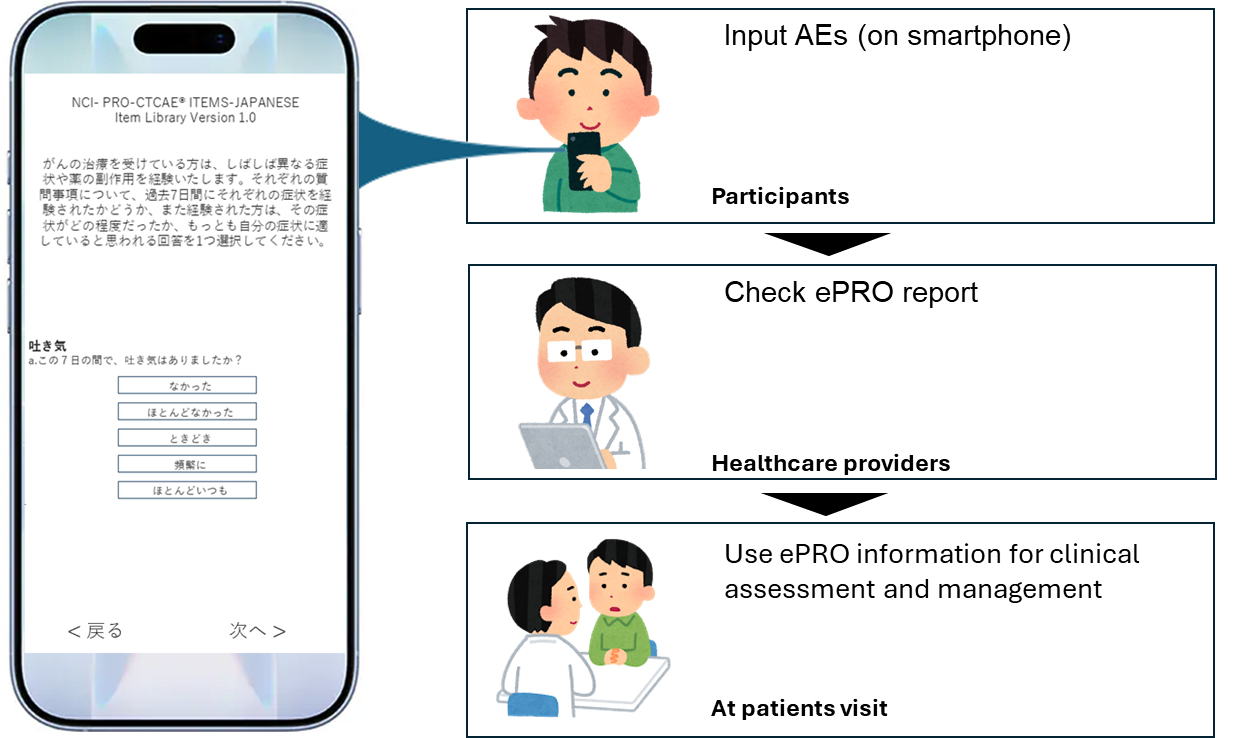


Participants entered adverse events (AEs) via weekly PRO-CTCAE/CTCAE questionnaires on their own smartphones. ePRO responses were aggregated into reports, which were made available to healthcare providers. At outpatient visits, clinicians were expected to review this ePRO information to support symptom assessment and treatment management.

**Supplementary Figure S3.** Distribution of the total number of alerts per patient over 12 weeks.


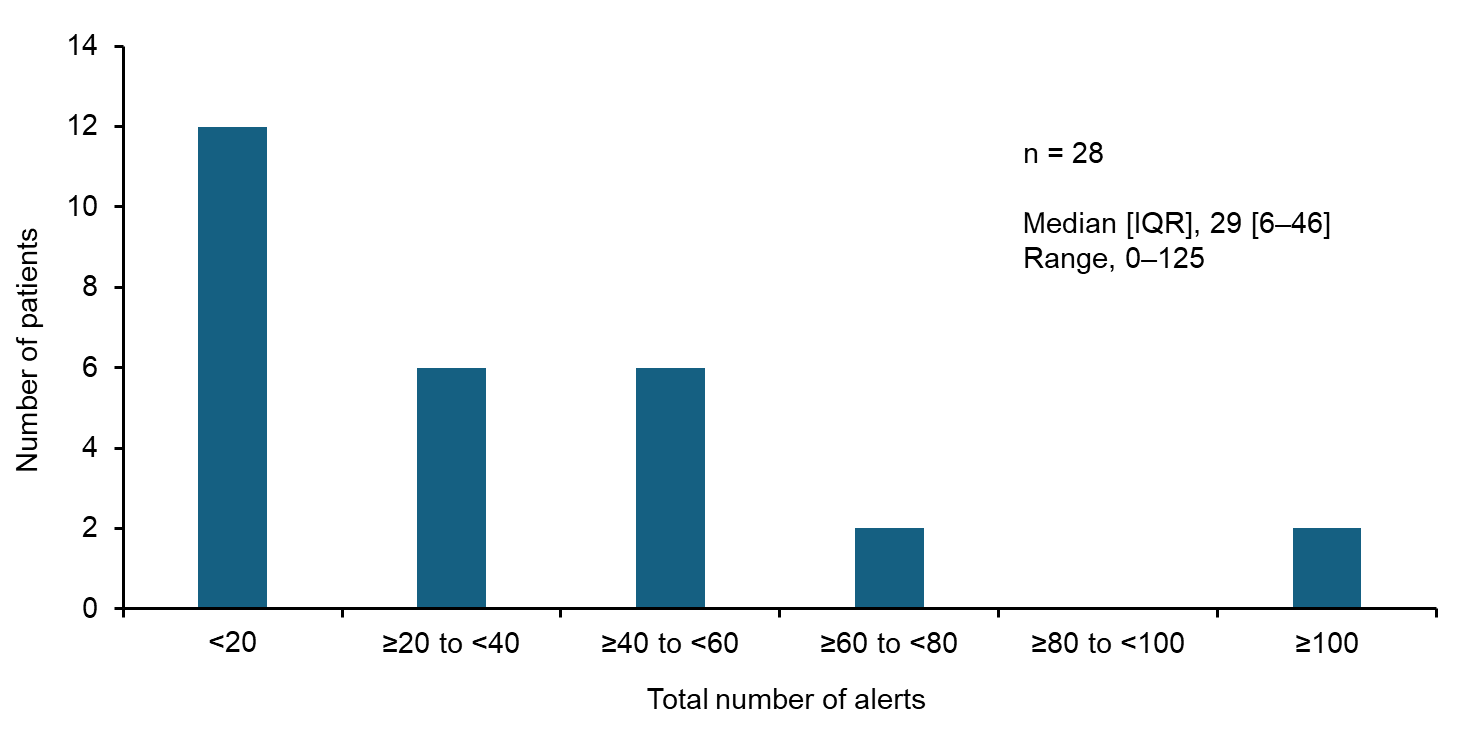


An “alert” was defined as an alert-triggering item in a completed weekly questionnaire (grade ≥3 for severity/frequency items or “present” for binary items).
